# A Mechanism-Based Approach to Prevention of and Therapy for Fibromyalgia

**DOI:** 10.1155/2012/951354

**Published:** 2011-10-02

**Authors:** Charles J. Vierck

**Affiliations:** Department of Neuroscience and Comprehensive Center for Pain Research, Colleges of Medicine and Dentistry, University of Florida, P.O. Box 100444, 1600 S.W. Archer Road, Gainesville, FL 32610, USA

## Abstract

Fibromyalgia syndrome (FMS) is characterized by pain referred to deep tissues. Diagnosis and treatment of FMS are complicated by a variable coexistence with regional pain, fatigue, sleep disruption, difficulty with mentation, and depression. The widespread, deep pain of FMS can be a consequence of chronic psychological stress with autonomic dysregulation. Stress acts centrally to facilitate pain and acts peripherally, via sympathetic vasoconstriction, to establish painful muscular ischemia. FMS pain, with or without a coexistent regional pain condition, is stressful, setting up a vicious circle of reciprocal interaction. Also, stress interacts reciprocally with systems of control over depression, mentation, and sleep, establishing FMS as a multiple-system disorder. Thus, stress and the ischemic pain it generates are fundamental to the multiple disorders of FMS, and a therapeutic procedure that attenuates stress and peripheral vasoconstriction should be highly beneficial for FMS. Physical exercise has been shown to counteract peripheral vasoconstriction and to attenuate stress, depression, and fatigue and improve mentation and sleep quality. Thus, exercise can interrupt the reciprocal interactions between psychological stress and each of the multiple-system disorders of FMS. The large literature supporting these conclusions indicates that exercise should be considered strongly as a first-line approach to FMS therapy.

## 1. Mechanistic Base of FMS Prevention and Therapy

Clinical diagnosis of fibromyalgia syndrome (FMS) has relied heavily upon tender point counts, a convenient evaluation of pain sensitivity that has come under scrutiny in terms of reliability and validity [1]. Tender point testing is designed to provide objective evidence for hypersensitivity to palpation of deep tissues, consistent with patient reports of ongoing pain referred to deep tissues. The location of ongoing pain changes over time but is widespread in the aggregate. The pain is chronic but is not always present. These features suggest that deep tissues are chronically sensitized and are easily brought to threshold for activation of nociceptors. Accordingly, tests of deep pressure sensitivity with control over the stimulus and thorough psychophysical evaluations reveal allodynia and hyperalgesia for stimulation of a muscle within the aggregate distribution of FMS pain. When muscular indentations are controlled in duration and force, FMS subjects report lower pain thresholds [2] and substantially more pain for suprathreshold stimulation than control subjects [2–8]. Repetitive stimulation at the threshold force for pain during a single indentation produces higher ratings by FMS subjects, compared to repetitive threshold stimulation for control subjects. Pain is longer in duration for FMS subjects following a series of repetitive muscular indentations. These observations [8] provide diagnostic verification of the ongoing deep muscular pain that brings FMS patients to the clinic.

FMS pain can arise from peripheral influences of the autonomic nervous system [9, 10] in response to aversive mood states (e.g., anxiety, fear, sorrow, and depression) that are referred to generally as mental suffering or distress. Distress activates the hypothalamopituitary axis (HPA) and sympathetic nervous system to generate physiological adaptations referred to as psychological stress reactions [11]. Stress, when chronic, results in cardiovascular dysfunction [12–16] with reduced peripheral blood flow as a result of vasoconstriction [17–19] and reduced endothelium-dependent vasodilatation [20, 21]. Also, chronic stress produces mitochondrial damage and pathological changes in the vasculature that reduce blood flow [22]. Resting levels of peripheral vasoconstriction are particularly high for females, relative to males [23–29], and FMS primarily is a female pain disorder [10]. Consistent with a predisposition toward peripheral vasoconstriction, females are particularly susceptible to Raynaud's syndrome [30, 31], characterized by excessive cutaneous vasoconstriction and pain in response to ambient cold. Also, peripheral artery disease (PAD) with reduced peripheral blood flow is more prevalent for women [32, 33]. Over time, reduced peripheral blood flow can lead to development of muscular ischemia [34, 35].

Phasic peripheral vasoconstriction, when robust, is painful, as demonstrated by the cold pressor test. Recordings of muscle nerve sympathetic activity (MSA) during ice water immersion of a hand or foot have shown that pain and MSA activity are highly correlated [36]. Similarly, acute muscular ischemia is painful, as evidenced by the submaximal effort tourniquet test [37]. Thus, when psychological stress and peripheral vasoconstriction become chronic and establish muscular ischemia, with sensitization of nociceptors [38, 39], muscular pain is easily evoked [9]. In turn, nociceptive input (e.g., from ischemic muscles) increases sympathetic vasoconstrictor outflow to muscles [40, 41], reinforcing the ischemia. Pain from vasoconstriction and muscular ischemia can explain the referral of FMS pain to deep tissues. 

In addition to ongoing or spontaneous muscular pain, psychophysical testing of FMS individuals has revealed widespread cutaneous hyperalgesia [42]. A parsimonious explanation for this effect is that nociceptive input from deep tissues sensitizes spinal neurons having convergent input from the skin, resulting in cutaneous hyperalgesia within the distribution of deep FMS pain [43]. The most thoroughly studied form of central sensitization is temporal summation (windup), a form of central synaptic magnification that requires repetitive or tonic nociceptive input to central neurons [43]. This phenomenology has led to proposals that central sensitization underlies FMS pain [42] and also is responsible for the widespread cutaneous hyperalgesia that accompanies regional pain conditions [44]. However, central sensitization from nociceptive driving does not readily explain the widespread cutaneous hyperalgesia that accompanies regionally localized pain conditions [37, 45–59]. Spinal neurons supplying areas of cutaneous hypersensitivity can be located distant from a source of regional pain. For example, central nociceptive pathways from the foot and the face originate separately from the spinal cord and brain stem, with different thalamic relays to the cerebral cortex. In spite of this separation, temporomandibular pain is associated with enhanced sensitivity to nociceptive stimulation of the foot [56], and patients with irritable bowel syndrome are hypersensitive to stimulation of the face [54] (J. Riley, A. Mauderli, and C. Vierck, unpublished observations). Thus, a source of facilitation other than direct synaptic driving by nociceptive input (e.g., temporal summation) is required to generate widespread cutaneous hyperalgesia from a regional source of pain. The stress and autonomic dysregulation that accompany localized chronic pain can account for widespread hyperalgesia [58, 60–66, 66–68] and development of FMS pain after onset of a regional pain condition [50].

## 2. Status of FMS Muscles

The relationships outlined above indicate that a primary objective of FMS prevention and therapy should be to reduce sympathetic vasoconstriction and muscular ischemia, resulting in a loss or reduction of widespread deep pain and hyperalgesia. Prevention applies to the development of FMS in association with chronic stress (e.g., from a regional pain condition), and reducing stress and increasing blood supply to peripheral tissues should be therapeutic if FMS has developed. The need to increase blood flow to peripheral tissues is strongly supported by demonstrations of muscle pathophysiology among FMS patients. Microcirculation is deficient for FMS individuals, as indicated by reductions in capillary density, capillary permeability, and blood flow, resulting in low tissue oxygenation [69–73]. The normal increase in blood flow during dynamic and static exercise (hyperaemia) is attenuated for FMS subjects [74]. Intramuscular concentrations of pyruvate and lactate are increased for subjects with FMS and are negatively correlated with pressure pain thresholds (PPTs) for muscle [2]. Expression of genes that detect muscle metabolites signaling pain and fatigue is increased following exercise by FMS individuals [75]. The strength and endurance of FMS subjects is decreased and is associated with high ratings of exercise induced pain [76, 77]. During and following dynamic exercise, muscle tension of FMS subjects is increased [76]. Inflammatory activity is altered toward an overproduction of proinflammatory cytokines [77–82]. Mitochondrial dysfunction of FMS individuals has been described, with a CoQ_10_ deficiency in blood mononuclear cells, increased oxidative stress, and mitophagy [83]. Thus, peripheral pathology associated with chronically reduced blood flow to deep tissues clearly can be a factor in generation of FMS pain. 

## 3. Exercise Effects on Muscles

Long-term programs of exercise have beneficial effects on stress and its effects on muscles of individuals with FMS and other conditions of ischemic muscular pain. Cardiovascular consequences of acute stress have been shown to be attenuated following a bout of exercise [17, 84–88]. For individuals with hypertension, a condition associated with chronic stress, exercise decreases sympathetic output to muscles [89] and decreases peripheral vascular resistance [90]. Thus, sympathetic activation by acute or chronic stress can be attenuated by exercise. Exercise promotes angiogenesis and attenuates symptoms of intermittent claudication and ischemic muscle pain associated with peripheral artery disease [91]. Muscular contraction induces secretion of vascular endothelial growth factor (VEGF), an essential contributor to capillary growth in skeletal muscles [92]. Accordingly, capillary density is increased by long-term exercise programs [93, 94]. Exercise increases expression of genes involved in mitochondrial biogenesis [95]. Also, oxygen consumption (VO_2_) is increased during exercise, to meet increased energy needs, and it remains elevated for hours following exercise—particularly when the exercise is distributed in time [96]. Blood flow to an exercised muscle is increased [97–100], along with VO_2_ [101], with little generalization to uninvolved muscles, avoiding hypotension [102]. The increase in blood flow in proportion to the relative activity of different muscle groups is referred to as functional sympatholysis, which declines with age [103]. Also following exercise, there is generalized sympathoinhibition [104] and prevention or reversal of endothelial dysfunction [105, 106]. Important functions of the endothelium that are enhanced by exercise include vasodilation, regulation of neovascular growth, and inflammatory control [105, 107].

## 4. Temperature Regulation and Blood Flow to Muscles

Control over blood flow to peripheral tissues is a fundamental component of temperature regulation. Cold environmental temperatures generate vasoconstriction, and warm temperatures elicit vasodilation, resulting in heat retention or loss, respectively [108]. The implications of temperature regulation for FMS are obvious. Individuals with FMS should stay warm, and exercise generates body heat. Studies of sauna heat therapy have revealed significant improvement in blood flow (endothelium-dependent dilation) [109–111] and reductions in FMS pain [112, 113]. Exercise in warm water is an effective therapeutic procedure for FMS [114], with long-term reductions in pain [115]. Exercise in water is well tolerated by FMS individuals, as it limits stress on weight bearing joints and provides resistance in proportion to the speed of movements.

## 5. Exercise Therapy for Distress and Depression

In addition to widespread pain and hyperalgesia, FMS is associated with disrupted control over numerous physiological and psychological functions. Accordingly, there has been a shift in emphasis away from seeing FMS strictly as a pain disorder toward regarding it as a multisystems disorder [1, 116]. Symptoms frequently associated with FMS pain include sleep disruption, depression, fatigue, and altered mentation (fibrofog). Mechanistically, these multiple symptoms of FMS can be seen as products of FMS pain and the stress inevitably associated with pain. Sleep disruption, inactivity, and fatigue are predictable consequences of chronic pain [117] and stress [118–120]. Similarly, depression [118, 121, 122] and memory disturbances [123, 124] can result from chronic stress. Thus, FMS fundamentally is a disorder involving reciprocal interactions between pain and stress. Pain can result from or be enhanced by chronic stress [49, 66, 125–132], and pain produces stress [64, 133–137], which has widespread influences on biological systems [11, 62]. 

Mood disorders both elicit stress and are consequences of stress. Distress, the driving force for chronic psychological stress with HPA and sympathetic activation, is attenuated by exercise programs for individuals relatively free of autonomic dysregulation [138] or with hypertension [139] or chronic pain [140, 141]. Depression, a mood disorder which often accompanies chronic pain, including FMS, is associated with autonomic dysregulation [142] and with cardiovascular disease [143]. Depression can evolve from chronic stress [121, 144, 145] and from chronic pain [146, 147], and it is a risk factor for chronic pain [142, 148, 149]. Stress, chronic pain and depression frequently coexist and are considered to be reciprocally related [150], each facilitating the other. As depression increases, so do pain complaints, and as pain episodes increase in intensity, frequency, duration, or variety, depression becomes more likely [151]. Exercise can disrupt these interactions by reducing pain [114, 140, 141, 152–156] and depression [114, 140, 141, 152, 153, 156–159]. Conversely, depression occurs more frequently for sedentary individuals [160] or when a long-term program of exercise is interrupted for as little as 2 weeks [161].

## 6. Stress, Exercise, and Mentation

Psychological stress can either facilitate or interfere with learning and memory, depending upon the timing of HPA activation relative to the event to be learned or remembered, and the magnitude and duration of stress are critical variables [124, 162]. While acute stress can be beneficial, chronic stress is detrimental to learning and memory. Activation of the HPA by psychological stress prominently involves the prefrontal cortex, amygdale, and hippocampus, with feedback regulation via corticosteroid receptors in these structures [123]. Chronic activation of corticosteroid receptors within the prefrontal cortex results in neuronal damage, impairing learning and memory [163, 164]. The hippocampus is a component of the cerebral circuitry mediating psychological stress and is a crucial structure for memory consolidation. Stress reduces neurogenesis in the hippocampus [165, 166], impairing memory and contributing to the pathophysiology of depression [167]. Depression and cognitive decline are linked phenomena [168]. 

Beneficial effects of exercise on mentation have been documented thoroughly [169]. Large surveys have revealed an inverse relationship between cognitive decline (including Alzheimer's dementia) and levels of exercise. Prospective studies have similarly shown an inverse relationship between objective fitness measures and cognitive decline. The largest effect sizes were for executive functions such as planning, working memory, and multitasking. Investigations of exercise programs for individuals with dementia have revealed beneficial effects on cognitive tests, increased cerebral blood flow, and spared brain volume. A dose-response relationship pertains to exercise duration/intensity and quality of life for older individuals [170].

Laboratory animal experiments have revealed mechanisms for exercise effects on mentation [168]. In brief, exercise induces a cascade of growth factor signaling that enhances cognitive function and attenuates depression by stimulating neuroplasticity and neurogenesis and improving blood flow. The growth factors IGF-1, BDNF, and VEGF are increased peripherally and centrally by exercise. IGF-1 increases BDNF signaling in response to exercise, enhancing neurogenesis and synaptic plasticity in the hippocampus and thereby facilitating learning and memory. Peripheral IGF-1 and VEGF are necessary for exercise-induced prevention of peripheral risk factors for cognitive decline, such as hypertension, hyperglycemia and inflammation.

## 7. Stress, Exercise, and Sleep

Psychological stress, pain, and sleep disruption are reciprocally related. Stress increases pain sensitivity [60, 66, 67, 171] and disrupts sleep [120, 172]; sleep disruption results in stress [173] with increased pain sensitivity [117, 174, 175]; pain produces stress [58, 61–64, 66, 68] and sleep disruption [117, 175–178]. Given the beneficial effects of exercise on stress and pain, reviewed above, it is not surprising that exercise has been reported to improve sleep latency, quality, efficiency, and duration and reduce next day tiredness [178–184].

## 8. Methodological Considerations for Prevention of and Therapy for FMS Pain

Ideally, a preventative/therapeutic regimen would not only have beneficial effects on stress, autonomic dysregulation, and pain but would directly or indirectly attenuate the multisystem aspects of FMS such as sleep disruption, depression, fatigue, and fibrofog. Exercise has the potential to accomplish these goals, but it is difficult to convince an FMS patient that an acutely painful activity will reduce pain in the long run. FMS pain can be increased during a bout of exercise [77, 185, 186], and pain sensitivity can be increased at the conclusion of exercise [77, 187]. Peripheral receptors that contribute to muscular pain and fatigue [38] are expressed and detected in leukocytes following exercise by individuals with FMS [75]. These effects are to be expected during and after working ischemic muscles. However, despite the barrier of activity-induced pain, carefully structured exercise programs can attenuate FMS pain and associated symptoms. Gradual introduction to an exercise protocol is especially important for FMS patients with chronic fatigue syndrome (CFS), for whom exertion can cause postexercise malaise for several days.

Numerous studies of exercise effects on FMS symptoms have been summarized in meta-analysis reviews that critically evaluate the experimental methods and summarize the results. The benefits of mild to moderate exercise programs for FMS uniformly include enhanced well being, improved physical fitness, and reduced disability [140, 141]. Because deconditioning commonly accompanies FMS [65], a strong case can be made for exercise as a standard component of FMS therapy. Furthermore, pain and/or tender point counts clearly are decreased by most exercise paradigms [114, 140, 141, 152–156]. The success rate of exercise for pain is important, relative to therapies that rely exclusively on pharmacological agents with side effects and inherent difficulties associated with long-term usage [156]. However, individual differences in response to exercise result in a moderate overall (average) effect on FMS pain [141, 154, 156, 188]. Individual variability is considerable with respect to the severity, duration, and variety of FMS symptoms at the inception of treatment, and the duration of chronic pain is an important consideration. Also, the type and frequency of exercise and long-term compliance of the subjects influence the benefits of exercise therapy. 

Typical experimental protocols for aerobic exercise have conducted supervised sessions 2 or 3 times per week [153–155], with varying recommendations for and documentation of exercise at home. Low to moderate levels of aerobic exercise, as defined by heart rate and blood pressure recordings, have been recommended [152–155]. Direct comparisons with strength training or stretching exercises have concluded that aerobic procedures provide better results [141, 154]. The reasons for this conclusion are not clear, because assessments of presumed mechanistic bases for FMS are not included. For example, what are the relative effects of aerobic and strength exercises on indices of stress, autonomic regulation, and peripheral blood flow, and how are these effects related to reduction of deep muscular pain? In terms of peripheral mechanisms of FMS such as widespread muscular ischemia, it seems that an exercise protocol should enhance peripheral circulation globally, rather than for a specific set of muscles. This goal may be met by whole body aerobic exercise or strength training of multiple muscle groups, but documentation is needed. Also, summaries of exercise therapy for FMS have strongly recommended tailoring of parameters of exertion for each patient [153, 154, 156, 188]. More specifically, (1) a level of exercise which is painful for a subject will discourage participation and may be deleterious [188]; (2) it is important to have exercise options that are adaptable to frequent use by individuals with different daily routines and/or physical limitations; (3) because chronic stress and autonomic dysregulation are relentless, exercise routines are likely to be most effective if conducted frequently so that peripheral blood flow is increased over a significant portion of each day. 

The optimal schedule of exercise likely will depend upon the duration of increased blood flow that can be expected to accompany and follow each exercise period. Exercise training appears not to affect muscle blood flow at rest [189]. Thus, exercise therapy for FMS is at an important juncture, needing thorough investigations of long-term effects of different forms of exercise on blood supply to deep tissues. It is encouraging that studies involving standardized schedules of infrequent exercise have revealed attenuation of FMS symptoms, but there is little to be gained by continued study of set exercise paradigms with a fixed set of parameters that are chosen arbitrarily. 

The optimum benefits of exercise for FMS surely will depend upon repeated, daily periods of exertion, but regularly scheduled laboratory sessions will be necessary to evaluate and adjust paradigms of exercise. Tolerance for exercise (exercise induced pain) can be assessed, both to maximize continued participation and to measure beneficial effects of exercise. If peripheral blood flow is increased over time with exercise, the threshold for exercise-induced pain should increase. Also, there should be a period free of clinical pain following each exercise period [186], and charting the time-course of this effect in relation to changes in blood flow to deep tissues would be instructive for design and modification of the exercise protocol. Resting levels of blood flow to muscles, as well as changes in blood flow in response to exercise or nociceptive stimulation, are regarded here as crucial information. 

Previous studies have relied upon tender point counts as a measure of deep pain sensitivity, but a psychophysical test of pain during and after well-controlled compression of several muscle groups can provide more useful information on effects of exercise, over time, on sensitization of nociceptors in deep tissues. Psychophysical tests of sensitivity to cutaneous stimulation (e.g., temporal summation during repetitive stimulation) provide information on the central sensitization that is driven by tonic nociceptive input. Techniques for detection of biomarkers of gene expression have been shown to be particularly informative concerning levels of receptor expression and immune activation that are associated with muscular fatigue and pain [75, 190]. These means for evaluation of mechanisms have provided opportunities to approach FMS as a medical condition. Until recently, there were questions as to whether FMS is psychosomatic, without an identifiable organic basis, in contrast to regional pain conditions with histories of injury to the painful tissues. Ironically, the mechanistic bases of FMS appear be identifiable and may also be correctable, in contrast to many regional pain conditions without available therapies that can silence the source of nociceptive input.

## 9. Summary and Therapeutic Implications

Fibromyalgia is a multiple-system disorder. FMS patients complain of chronic pain referred to deep tissues (and pain from exercise or palpation of muscles) and commonly present with depression, fibrofog, and sleep disruption. FMS patients frequently are in a deconditioned state with fatigue, and the widespread deep pain of FMS often coexists with one or more regional pain conditions. It is not feasible to treat each of these disorders separately (e.g., with pharmacological agents directed specifically to treat each disorder). This conundrum forces consideration of whether there is a fundamental mechanism for the symptoms that define FMS as a multiple-system disorder. Evidence summarized here identifies chronic psychological stress with autonomic dysregulation as the root cause of FMS, providing opportunities for mechanism-based prevention and therapy [191]. Pain referred to deep tissues is considered the primary symptom of FMS. Chronic stress reduces peripheral blood flow, resulting in widespread muscular ischemia, and muscular pain is a powerful stressor. Stress, with autonomic dysregulation and pain, also establishes central influences that enhance depression, impair mentation and sleep, and increase pain. Therefore, a therapy that reduces stress and/or pain could alleviate each of the multiple-system disorders of FMS. Remarkably, exercise exerts beneficial effects on stress and pain and the other FMS disorders. Reports documenting these effects are part of an extensive literature providing evidence for exercise as an effective therapy for many chronic diseases of a deconditioned modern society [192].

Investigations typically report moderate overall effects of exercise on FMS symptoms, attributable to the use of standardized exercise protocols despite considerable variability between patients. The use of fixed exercise protocols appears to suit scientific purposes but conflicts with a necessity to tailor exercise to each individual for the maximal therapeutic effect. Thus, it is recommended that different forms, frequencies, durations, and intensities of exercise be evaluated in terms of sustained normalization of peripheral blood flow for FMS individuals. Once an optimal pattern of exercise is established, it can be utilized with measurements of peripheral blood flow to guide individual variations in the exercise protocol over time. It can be expected that effective exercise protocols for pain reduction will differ between subjects and over time, as dictated by blood flow to muscles. 

A mechanistic approach to FMS therapy and research minimizes the importance of a control group for comparison with treatments such as exercise. The therapeutic goal is to alleviate FMS, and important scientific goals are to evaluate relationships between FMS and suspected biological mechanisms for the FMS symptoms. At this point, these comparisons can be more instructive than group comparisons with and without exercise. For evaluation of FMS pain, it will be important to evaluate elicited ischemic pain in addition to standard verbal reports of ongoing pain. Well-controlled pressure stimulation of muscles with psychophysical evaluation of pain threshold and the suprathreshold intensity and duration of muscular pain is more informative than pressure point counts. Measurements of peripheral blood flow and exercise-induced expression of biomarkers for receptor expression and immune activation [75] are informative concerning peripheral effects of exercise. Assessments of distress, depression, sleep quality, and mentation provide a tracking of central effects of exercise. 

Correction of deconditioning with exercise is beneficial for FMS patients, but available evidence indicates that psychological stress and peripheral vasoconstriction must be attenuated to alleviate pain referred to deep tissues. This paper has not covered techniques to relieve psychological stress directly, but they can be effective in combination with exercise, attenuating reciprocal interactions with pain and each of the multiple-system disorders of FMS.
